# German Cranial Reconstruction Registry (GCRR): protocol for a prospective, multicentre, open registry

**DOI:** 10.1136/bmjopen-2015-009273

**Published:** 2015-09-30

**Authors:** Henrik Giese, Thomas Sauvigny, Oliver W Sakowitz, Michael Bierschneider, Erdem Güresir, Christian Henker, Julius Höhne, Dirk Lindner, Dorothee Mielke, Robert Pannewitz, Veit Rohde, Martin Scholz, Patrick Schuss, Jan Regelsberger

**Affiliations:** 1 Department of Neurosurgery, University of Heidelberg, Heidelberg, Germany; 2 Department of Neurological Surgery, University Medical Center Hamburg-Eppendorf, Hamburg, Germany; 3 Department of Neurosurgery, Klinikum Ludwigsburg, University of Heidelberg, Ludwigsburg, Germany; 4 Department of Neurosurgery, Trauma Center Murnau, Murnau, Germany; 5 Department of Neurosurgery, University Hospital Bonn, Bonn, Germany; 6 Department of Neurosurgery, University Hospital Rostock, Rostock, Germany; 7 Department of Neurosurgery, University Hospital Regensburg, Regensburg, Germany; 8 Department of Neurosurgery, University Hospital Leipzig, Leipzig, Germany; 9 Department of Neurosurgery, University Medical Center Göttingen, Göttingen, Germany; 10 Department of Neurosurgery, Heinrich-Heine-University, Düsseldorf, Germany; 11 Department of Neurosurgery, Duisburg Medical Center, Duisburg, Germany

**Keywords:** NEUROSURGERY

## Abstract

**Introduction:**

Owing to increasing numbers of decompressive craniectomies in patients with malignant middle cerebral artery infarction, cranioplastic surgery becomes more relevant. However, the current literature mainly consists of retrospective single-centre (evidence class III) studies. This leads to a wide variability of technical approaches and clinical outcomes. To improve our knowledge about the key elements of cranioplasty, which may help optimising clinical treatment and long-term outcome, a prospective multicentre registry across Germany, Austria and Switzerland will be established.

**Methods:**

All patients undergoing cranioplastic surgery in participating centres will be invited to join the registry. Technical methods, materials, medical history, adverse events and clinical outcome measures, including modified Rankin scale and EQ-5D, will be assessed at several time points. Patients will be accessible to inclusion either at initial decompressive surgery or when cranioplasty is planned. Scheduled monitoring will be carried out at time of inclusion and subsequently at time of discharge, if any readmission is necessary, and at follow-up presentation. Cosmetic results and patient satisfaction will also be assessed. Collected data will be managed and statistically analysed by an independent biometric institute. The primary endpoint will be mortality, need for operative revision and neurological status at 3 months following cranioplasty.

**Ethics and dissemination:**

Ethics approval was obtained at all participating centres. The registry will provide reliable prospective evidence on surgical techniques, used materials, adverse events and functional outcome, to optimise patient treatment. We expect this study to give new insights in the treatment of skull defects and to provide a basis for future evidence-based therapy regarding cranioplastic surgery.

**Trial registration number:**

This trial is indexed in the German Clinical Trials Register (DRKS-ID: DRKS00007931). The Universal Trial Number (UTN) is U1111-1168-7425.

**Strengths and limitations of this study**

- The German Cranial Reconstruction Registry (GCRR) is a prospectively conducted, multicentre, international, open registry study in which patients will be observed long term.
- Owing to the prospective international nature (Germany, Austria, Switzerland), a very wide range of cranioplastic treatment options will be detected. Similar publications and studies are only monocentric.
- The registry includes patients with decompressive craniectomies in a wide range of acute diseases (eg, traumatic brain injuries, space-occupying cerebral infarction, subarachnoid and intracerebral haemorrhage, etc) as well as destructive or osteolytic bone tumours.
- A limitation of the study is that the GCRR is not a randomised study, but only a pure observational study. Specific treatment strategies will not be compared against each other.
- One of the strengths as well as simultaneous limitation of this study is the planned long observation period of up to 10 years. There is the possibility of a high number of participants lost to follow-up.

## Introduction

Several prospective clinical trials have demonstrated that early decompressive craniectomy (DC) increases survival in patients with space-occupying middle cerebral artery (MCA) infarction by reducing intracranial pressure. Subsequently, numbers of DCs are continuously rising.^1–5^ Large cerebral infarctions cause space-occupying brain oedema, which is considered the main reason for a mortality rate of up to 80%.^6–8^ Despite best conservative treatment, outcome was generally poor before establishing DC as an early treatment option.^6^ Recently, analyses of prospective randomised clinical trials including the DECIMAL (Decompressive Craniectomy In Malignant MCA Infarction), HAMLET (Hemicraniectomy After Middle cerebral artery infarction with Life-threatening Edema Trial), DESTINY I and DESTINY II (Decompressive Surgery for the Treatment of malignant Infarction of the middle cerebral artery) trials, and a prospective pooled data analysis of the first three trials mentioned above, proved the benefit for patients with malignant MCA infarction.^2–4^ ^9–11^ The probability of survival for patients aged 60 years and younger increases up to 80% following DC (vs 28% without DC) and the probability of survival with a modified Rankin scale (mRS) of ≤3 doubles.^2^ Based on these beneficial experiences, DC procedures have also been proposed as a therapeutic option in refractory intracranial hypertension or malignant brain oedema due to traumatic brain injury (TBI), acute subdural haematoma, subarachnoidal haemorrhage or encephalitis.^12–17^

Each successful DC requires a secondary operative procedure: cranioplasty (CP). Besides refitting the integrity of the skull, protecting the brain and restoring the cosmetic aspect, CPs have gained a major focus in the rehabilitation process. CPs may also improve the neurological condition of patients with a so-called ‘sinking skin flap syndrome’, and associated deteriorations, dramatically.^18^ Furthermore, CP may play an important role in reconstruction procedures following excision of primary osseous tumours, meningiomas infiltrating the bone and simple removal of bone flaps following postoperative infection.

Both DC and CP are performed with increasing frequency, while published series reveal a 20–50% complication rate. Complications following DC after malignant infarction include haematomas, meningitis, seizures and wound infections.^19^ Major complications of DC after severe TBI are herniation of the brain tissue through defects (34%), subdural effusions (54%) and hydrocephalus (14%).^20^ One-third of all patients undergoing CP suffer from complications,^21–23^ of which wound infections and wound healing disorders, in up to 25% of patients, are of major importance, as is aseptic bone necrosis, in up to 18%.^21^ ^24^ Although some predictors, such as multiple fractures within the bone flap, wound infection after CP and insecure fixation of the bone, may increase bone resorption rates, the impact of other factors, such as bone flap preservation or timing of CP, is unknown.^21^ ^25^ In the absence of prospective clinical studies, and based on numerous retrospective monocentre studies alone, one cannot reach consensus regarding timing of CPs, materials and perioperative management.^21^ ^23^ ^26–29^

The aims of this international prospective multicentre register are to (1) identify surgical and medical factors with a strong influence on patient's outcome and functional status, and (2) establish an evidence-based therapeutic approach optimising timing and the procedures themselves, thus minimising perioperative and postoperative complication rates and improving clinical outcome in this group of patients.

## Methods

### Study design

The German Cranial Reconstruction Registry (GCRR) is a prospectively conducted, multicentre, open registry, in which patients will be observed long term. The study is a procedure-specific registry, initiated by a consortium of individual members of the Section for Neurotrauma and Intensive Care in Neurosurgery of the Deutsche Gesellschaft für Neurochirurgie (DGNC). A total of 10 German neurosurgical departments have constituted a Steering Committee, which will be responsible for the scientific goals of the registry and guarantee the independence of the data analysis performed by a biometric institute. In 2014, organisation and goals of the registry as well as the implementation of the GCRR were determined. Neurosurgical units in Germany, Austria and Switzerland conducting DC and CPs are invited to join the registry and to recruit patients.

### Study setting and type of participants

All patients undergoing CP are included in the study. Inclusion criteria are all clinical conditions that require a temporary removal of the skull, for example: TBIs, space-occupying cerebral infarction and subarachnoid haemorrhage as well as destructive or osteolytic bone tumours. Patients undergoing craniectomy without CP (eg, suboccipital), craniosynostosis repair or skull base approaches with complex reconstruction, will not be included (table 1). Patients can be included at two time points: either at the initial event of DC or at readmission for CP.

**Table 1:** Inclusion and exclusion criteria for patients to participate or not in the GCRR

Inclusion criteria	Exclusion criteria
Patients with a clinical condition that requires *temporary* removal of the cranial bone (DC) Space-occupying cerebral infarction Traumatic brain injury Subarachnoid haemorrhage Intracranial haemorrhage Sinus venous thrombosis Space-occupying cerebral infections Patients after DC who now require surgical CP Patients with osteolytic or bone-destructing tumours of the skull Legal age (≥18 years)	Patients with a clinical condition that requires *permanent* removal of the cranial bone Patients in palliative care Patients with craniofacial malformations (eg, craniosynostosis) Patients who require skull base reconstruction Patients after suboccipital DC

CP, cranioplasty; DC, decompressive craniectomy; GCRR, German Cranial Reconstruction Registry.

### Informed consent

Written informed consent for this study will be obtained from the patient or the patient’s authorised representative prior to study inclusion. The study will be conducted in accordance with the provisions of the Declaration of Helsinki.

### Data collection

According to a standardised questionnaire for DC and CP, patient-specific data, risk factors, surgical details, materials for CP, and intraoperative and postoperative complications, will be recorded. Data acquisition will be paper-based (case report form, CRF), and patient-specific data will be anonymised by the study centre. The data will then be transferred to the Department of Medical Biometry Heidelberg (Institute of Medical Biometry and Informatics, IMBI) and transmitted into an electronic database. Despite anonymous data acquisition, patient tracking remains possible for analysing specific events such as clusters of complications or unexpected severe events.

### Case report form

**Figure 1:**
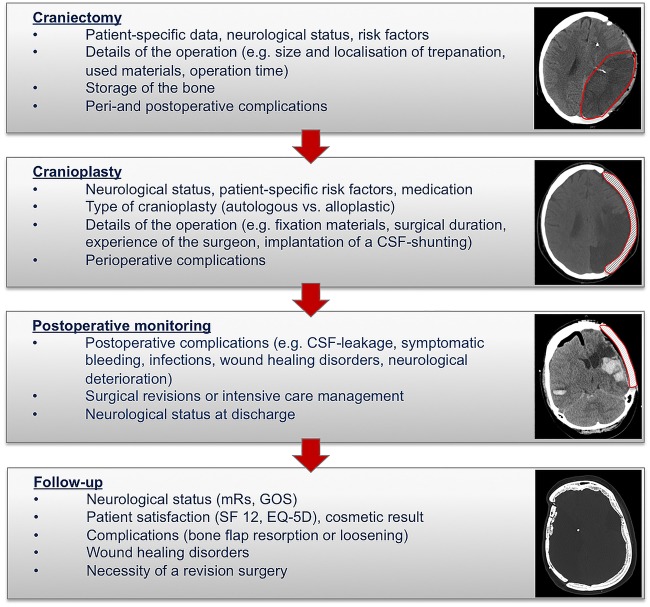
Study protocol of the German Cranial Reconstruction Registry (GCRR): using a structured questionnaire (for physicians and patients), patients’ clinical pathways will be monitored in four steps, from the craniectomy, over the cranioplasty and up to the long-term follow-up.

The specially designed and developed questionnaire (CRF) consists of four different parts (figure 1).

*Craniectomy*: The first part of the CRF covers the initial surgery, craniectomy. In addition to patient data (sex, age) and diagnosis, the initial neurological status (Glasgow Coma Scale (GCS), National Institute of Health Stroke Scale (NIHSS), World Federation of Neurosurgical Societies Scale (WFNS), Hunt and Hess), medical history (American Society of Anesthesiologists (ASA) Physical Status Classification), comorbidities (eg, diabetes, arterial hypertension, coagulopathy) and risk factors (eg, smoking status, drugs), is recorded. In addition, surgical data such as localisation of skin incision, trephination size and localisation, dura opening and closure technique (duraplasty) as well as materials used (eg, suture material and technique, drainage), are registered. Moreover, the experience of the surgeon, duration of operation, amount of blood loss and any perioperative antibiotic therapy given, will be documented. Finally, the storage of the bone and perioperative complications are listed. The initial observation period following DC (part 1) will end with discharge of the patient, where neurological status is recorded (Glasgow Outcome Scale (GOS), modified Ranking Scale (mRS), NIHSS).

*Cranioplasty*: The second part of the CRF covers all particular details of CP. Here, new patients can be included independently of the prior procedure, for example, when operated earlier at another institution. Again, preoperative data and the current neurological status are recorded. Particular attention is paid to the size of craniotomy and the type of CP (autologous vs alloplastic). All types of materials are included (eg, plastic, ceramic, titanium alloy, etc) and will be distinguished between patient-specific and hand-made CP. Details for fitting and fixating the material, simultaneous implantation of a CSF-shunt as well as standard parameters (suture material, size and number of drainage apparatus, perioperative antibiotics) will be covered. Similar to during the DC procedure, operating duration and amount of blood loss will be captured.

*Postoperative monitoring*: The third part addresses all questions of the clinical follow-up at time of discharge. Acute complications such as bleeding events, seizures, wound healing disorders, infections and CSF fistulas, will be recorded. Removal of drains and control images are documented as well if surgical revisions or intensive care surveillance and/or treatment have been required in case of any complications. On discharge, neurological status will be recorded (GOS, mRs) again. This CRF can be used for any readmission due to complications or further surgical treatment as well.

*Follow-up*: Long-term clinical outcome after 12 months will be recorded; this will be continued annually, and is covered by a fourth part. Here, long-term complications such as aseptic bone necrosis, loosening or displacement of CP, wound healing disorders and revision procedures, if required, are recorded. Similar to in the earlier step, neurological status (GOS, mRs), patient satisfaction (SF12, EQ-5D) and cosmetic result will be assessed.

### Data management

Data collection will be performed locally and anonymised for name and date of birth in an independent database, then submitted to the Department of Medical Biometry Heidelberg (IMBI). An interim analysis is planned after 2, 5 and 10 years, and it is estimated to include at least 80 patients per year. All results of the GCRR, including epidemiological data, surgical techniques, material for CP, complications, risk factors and long-term outcome, will be published and/or reported at respective scientific meetings. This study is indexed in the German Clinical Trials Register (DRKS-ID: DRKS00007931). The Universal Trial Number (UTN) is U1111–1168–7425.

### Statistics

Endpoints will be evaluated using descriptive statistics, and the key figures of the distributions will be presented in tables. Univariate analyses will allow for a first overview of potentially influential factors. Depending on the composition of the data, χ^2^, Mann-Whitney U and t tests, or Pearson or Spearman correlation coefficients, will be conducted. Relationships between multiple independent variables on the dependent variable(s) will be tested using multivariate regression analysis. Missing values will be replaced and estimated using multiple imputations. Furthermore, sensitivity analysis will be executed using complete-case analysis.

### Registry reports

Results of the GCRR will be published by the Steering Committee and distributed to all participating centres following careful analysis by the IMBI.

## Discussion

DC and CP are of increasing importance in neurosurgery. While they both are standard neurosurgical interventions, and are supposed to be ‘simple’ and ‘extracerebral’ surgical procedures, complication rates are surprisingly high and there is definitely a lack of evidence.

The GCRR is designed as a multicentre prospective data collection to uncover risk factors of both procedures, thus minimising complication rates, especially regarding periprocedural wound infections, CSF leakages and reasons for surgical revisions, as well as improving long-term clinical and cosmetic outcome focusing on CP materials, reasons for revision surgeries and rehabilitation processes. The amount of data and variety of aspects covered by this registry will give new insight in DC and CP, enhancing our knowledge regarding these ‘easy’ operative procedures. Currently, DESTINY-R is evaluating the short-term and long-term risks and benefits of DC in patients with MCA infarction in a large population routinely treated in neurological and neurosurgical units.^30^ The GCRR takes a step forward since it is not limited to one disease entity, and features a procedure and follow-up procedure in great detail. The effectiveness of registries and long-term surveillance of neurosurgical implants has been well demonstrated with, for example, the UK shunt registry.^31^ By the acquisition of nearly 33 000 CSF shunt-related procedures, for example, the benefit of antibiotic-coated catheters could be proven.^32^ Similarly, a registry for CP surgeries called ‘UK Cranial Reconstruction Registry (UKCRR)’ was founded for the UK.^33^ Both the GCRR and UKCRR have had overlap in the planning phase and contents of both registries were harmonised. Inspired by these successful registry projects, now the GCRR will be initiated for Germany, Austria and Switzerland.
